# Effect of in Planta Treatment of ‘Cavendish’ Banana with Herbicides and Fungicides on the Colonisation and Sporulation by *Fusarium oxysporum* f.sp. *cubense* Subtropical Race 4

**DOI:** 10.3390/jof7030184

**Published:** 2021-03-04

**Authors:** Jay Anderson, Elizabeth Aitken

**Affiliations:** 1 Centre for Organics Research, Southern Cross University, Lismore 2480, Australia; 2 School of Agriculture and Food Sciences, The University of Queensland, St Lucia 4072, Australia; e.aitken@uq.edu.au

**Keywords:** inoculum, epidemiology, *Fusarium oxysporum*, herbicide, biosecurity, Tropical Race 4 (TR4)

## Abstract

Fusarium wilt caused by the soil-borne fungus *Fusarium oxysporum* f.sp. *cubense* (*Foc*) is a significant constraint to banana production worldwide, with the recent expansion of banana growing regions impacted by *Foc* Tropical Race 4 (TR4). The lack of commercially acceptable Cavendish cultivars with *Foc* resistance means the only current means of effective control is through strict quarantine and inoculum management. One method of control that is currently advocated includes the removal of infected plants which have been killed using herbicide injections. The aim of this work was to examine the effect of herbicide and fungicide treatments on sporulation of the fungus. In glasshouse studies using a green fluorescent transformed *Foc* Subtropical Race 4 isolate, we found treatments with herbicide hastened colonisation of the banana tissue and the production of micro- and macroconidia. The use of a fungicide did not prevent sporulation of the fungus in such tissue. This study demonstrates that herbicide treated plants are a source of potential inoculum for infection of nearby plants.

## 1. Introduction

Fusarium wilt of banana caused by the soil-borne fungus *Fusarium oxysporum* f.sp. *cubense* (*Foc*) is one of the most devastating diseases of banana. The pathogen initially invades the roots and then colonises the vascular tissue of the plant resulting in wilting and eventual plant death [1]. *Foc* is divided into races based on the cultivars on which it causes disease. Banana cultivars are mostly clonal polyploids derived from either the A (*Musa acuminata*) or B (*M. balbisiana*) genomes or combination of both. Race 1 causes disease on ‘Gros Michel’ (AAA), ‘Maqueño’ (AAB), ‘Silk’ (AAB), ‘Pome’ (AAB) and ‘Pisang Awak’ (ABB) [1]. Race 2 affects ABB cooking banana whereas Race 4 affects most Race 1 and Race 2 susceptible cultivars in addition to Cavendish cultivars (AAA) [1]. Race 4 has been further divided into Subtropical Race 4 (SR4) causing disease on Cavendish cultivars in sub-optimal growing conditions, and Tropical Race 4 (TR4) which causes disease even when Cavendish plants are grown in optimal conditions [2]. A recent publication has placed TR4 into its own species, *F. odoratissimum* [3], however this is still yet to be widely adopted and the whole *F. oxysporum* species complex is under current revision [4].

The previous global epidemic of banana Fusarium wilt occurred when what is now known as *Foc* Race 1 caused extensive destruction to the ‘Gros Michel’ export plantations of Central America during the first half of the 20th Century. The resultant demise of the export industry led to, at that time, intensive research into the epidemiology and management of *Foc*. It was only the widespread adoption of Race 1 resistant Cavendish cultivars that allowed the export industry to survive and with this the necessity to study the epidemiology of the disease declined [2]. Since the early 2000s, TR4 has been detected at accelerating pace, including as of 2019 in Latin America and the Caribbean, the region of greatest export banana production being based on Cavendish [5]. This has reinvigorated research into the epidemiology and control of the disease in the absence of commercially acceptable resistant cultivars.

In Queensland, Australia, TR4 is under regulation and, where still-standing infected plants are detected, they must be injected with glyphosate prior to cutting down between 10 and 15 days after injection [6]. The pseudostem and leaf material are cut into 60–80 cm long pieces and sealed in plastic bags with urea, the corm is gouged out and urea is applied to the corm and surrounding area at a high rate [6]. The application of a high rate of urea to the soil and plant material is toxic to *Foc* [7]. Warman and Aitken [8] found that *Foc* SR4 sporulates readily on naturally senescing banana tissue producing chlamydospores in the gas spaces of leaf sheaths and sporodochia on the outside of leaf sheaths; it is unknown however if the fungus sporulates readily on banana plants which have been treated with herbicide.

Studies into the three-way interaction of herbicide, plant and *Fusarium oxysporum* have mostly examined the effect on disease development but not the potential production of inoculum, for example, the effect of herbicides on development of cotton wilt caused by *F.o.* f. sp. *vasinfectum* [9]. Work by Lèvesque et al. [10] examined the effect of glyphosate on the colonisation of weeds by *Fusarium* spp. The colonisation of the weeds was assessed by the re-isolation of the pathogen, but they also examined the effect on the number of propagules in the soil finding that treatment with glyphosate significantly increased the inoculum density. For effective disease management, there is a need to better understand the influence herbicide treatment has on colonisation and sporulation of *Fusarium oxysporum* in planta. Different herbicides have been found to impact in varying degrees on the development of fungi in vitro and in planta. For example, *Fusarium solani* f.sp. *phaseoli* exhibited a significant reduction in conidial germination in a solution of glyphosate, compared to solutions of lactofen and imazethapyr [11]. When experiments with the same pathosystem and herbicides were undertaken on plants in glasshouse experiments, re-isolation of *F.s.* f.sp. *phaseoli* was significantly higher from glyphosate and imazethapyr treated plants than from plants treated with lactofen [11]. In Australia, farmers have ready access to glyphosate, paraquat/diquat and atrazine hence these herbicides were included in our study.

Trials on fungicides have been undertaken to examine their ability to manage Fusarium wilt of banana. Nel et al. [12] found benomyl, propiconazole, cyproconazole/propiconazole and prochloraz significantly reduced Fusarium wilt symptoms in plants which had been root dipped in these fungicides prior to planting in infested soil. However, whether these fungicides can impact the potential inoculum load within infected plant material that has been destroyed or naturally decayed is unknown. Of benomyl, propiconazole, cyproconazole/propiconazole and prochloraz, only propiconazole is currently registered for field use in banana in Australia [13]. In addition, propiconazole, a fungicide which inhibits sterol biosynthesis in fungi [14], is xylem-mobile [15], hence likely to interact with *Foc* as it progresses through the xylem of banana plants [8].

The aim of this work was to understand the effect of treatments with herbicides and fungicides on the sporulation of *Foc* in planta. To enable visualisation of the fungus and as a marker to confirm Koch’s postulates, a green-fluorescent protein transformed isolate of *Foc* SR4 was used in these studies. Three separate experiments were conducted to examine treatment effects on the colonisation of banana plants by *Foc*: (1) the effect of different herbicides applied as pseudostem injections; (2) the effect of fungicide and herbicide foliar sprays and (3) the effect of fungicide drench in combination with foliar herbicide application.

It should be noted that the quarantine concerns of working with TR4 precluded its use in these studies.

## 2. Materials and Methods

### 2.1. Plants

Tissue culture grown ‘Williams’ Cavendish (Musa AAA) banana plants were used for all experiments. Plants were kindly supplied by Sharon Hamill, Department of Agriculture and Fisheries, Queensland. The tissue culture plants were de-flasked and planted into pasteurised UQ23 mix (70% composted pine bark, 30% coir) in 30-cell trays and maintained under a 12 h light and dark cycle for 4-6 weeks at room temperature. Plants were then potted into 200 mm pots, placed into a physical containment glasshouse and maintained at approximately 26 °C under natural day light conditions. When plants were suitably acclimatised to glasshouse conditions, at approximately 20 cm tall, they were inoculated.

### 2.2. Inoculum

*Fusarium oxysporum* f. sp. *cubense* (BRIP 23598, VCG 0120) SR4 had been previously transformed by Dr Leanne Forsyth to express green fluorescent protein (GFP) and hygromycin B resistance and the pathogenicity of the isolate confirmed [8]. Two different inoculation methods were used; either as an application of colonised millet (herbicide alone trial) or as root dip in conidial suspension (the two fungicide and herbicide trials). For the millet inoculation method, the millet was prepared as per the method of Warman and Aitken [8] except that the inoculum was applied as whole millet grains, not ground, and at a rate of 20mL colonised millet per pot. Once inoculated the millet was covered with potting mix and pots placed in plastic autoclave bags to prevent run-off. For non-inoculated control treatments, 20mL per pot of non-inoculated millet was applied in the same manner as for the inoculated.

For the conidial suspension inoculation, the GFP-transformed SR4 isolate (GFP *Foc*) was inoculated into potato dextrose broth (PDB) amended with hygromycin B and maintained on an orbital shaker for 6 days at approximately 24 °C, 130 rpm. Conidia were then harvested by filtering through sterile gauze, washed twice with sterile de-ionised water (SDW) and then the concentration adjusted to 1 × 10^6^ conidia/mL. Prior to inoculation, the plants were carefully removed from the pots and the roots were gently washed to remove the potting mix. The plants were then inoculated by dipping roots into the conidial suspension up to the crown for 30 min before being carefully repotted. Correspondingly, the non-inoculated controls were treated at the same time in the same manner except roots were submerged in SDW for 30 min. All plants were placed into plastic autoclave bags to prevent run-off of inoculum.

All experiments were carried out in physical containment laboratories and glasshouses under a notifiable low risk dealing as approved by the Australian Office of Gene Technology Regulator.

### 2.3. Fungicide and Herbicide Treatments

For each trial three single plants were subject to each treatment.

#### 2.3.1. Effect of Injection with Different Herbicides on Foc

For the herbicide injection trial using a range of products (Table 1), chemical treatments were applied 6 weeks after GFP *Foc* infested millet inoculation at the time when plants started to show external symptoms of Fusarium wilt (stem splitting). Injection with glyphosate is the current destruction protocol registered for TR4 infected plants in Australia [6]; an injection method based on this destruction protocol was developed to suit glasshouse grown plants. To inject plants, an 18-gauge needle was used to make a hole in the pseudostem at about 10 cm above the soil line. The needle was removed and the hole was then immediately injected with 1.6 mL of herbicide (Table 1).

Three weeks after treatment with the herbicides a plant from each treatment was harvested. Single plants from each treatment were then harvested 3.5 weeks and 7 weeks after treatment.

#### 2.3.2. Effect of Foliar Fungicide Application on Foc-Inoculated Plants Sprayed with Glyphosate

GFP *Foc* root dip inoculated plants were subjected to a combination of treatments with the fungicide propiconazole and the herbicide glyphosate as described in Table 2 starting 3 weeks after the conidial suspension with GFP *Foc* had been applied. The fungicide and herbicide treatments were applied using a hand-held atomizer, until just prior to run-off stage. Six weeks after the fungal inoculation, plants were harvested, sectioned and examined using confocal microscopy, isolations were also undertaken.

#### 2.3.3. Effect of Fungicide Drench Application on GFP Foc-Inoculated Plants Sprayed with Glyphosate

Eleven weeks after root dip inoculation, plants were subject to the fungicide and fungicide then herbicide treatment groups (Table 3). Fungicide treatments were applied as a drench around the base of each plant with 50 mL propiconazole (37.5 mL/L propiconazole, 105 g/L a.i.). Two weeks after the fungicide application, herbicide (15 mL/L of glyphosate, 360 g/L a.i.) was applied using a hand-held atomiser until just prior to run-off stage. Plant tissues were then sampled for microscopy (corm) and isolations (roots, corm and mid-rib of the throat) 3 weeks after herbicide treatment. At the time of sampling a hand lens was used to detect any colonisation visible on external plant parts, if present a slide was made to confirm if it was the GFP *Foc* which was present.

### 2.4. Examination of Samples and Re-Isolation of the Pathogen

Tissue from the corms and the midribs of leaves from the throat (area of new leaf emergence) (all three experiments) and roots (fungicide spray experiment) were hand sectioned, mounted in water and examined using Zeiss LSM700 Confocal Microscope using a 488 nm excitation laser with 555 nm emission. Whilst relatively large pieces of tissue could be examined (10 mm × 10 mm × 1 mm thick) it was not practical to examine all of the plant tissue from every plant, hence similar locations were sampled to give at least six sections per plant part within each experiment to gain representative samples to gain qualitative data.

Isolations were also performed from the roots (fungicide drench and spray trials only), corms and mid-rib tissue at plant throat height. Tissue portions (approx. 5 mm × 5 mm × 5 mm) were surface sterilised in 2% sodium hypochlorite for 1 min before being washed twice for 1 min in SDW. Portions were dried on sterile blotting paper and aseptically cut to smaller sections (approximately 2 mm × 2 mm × 2 mm) and plated into water agar (WA). Any fungi which grew out from the isolations were sub-cultured onto hygromycin-amended ¼ strength potato dextrose agar (HPDA) as well as a section of agar mounted on a slide and examined using the confocal microscope with 488 nm excitation laser to confirm re-isolation of the GFP *Foc*.

## 3. Results

### 3.1. Effect of Injection with Different Herbicides on Foc

Three weeks after herbicide treatment the outer leaf sheaths had collapsed on all inoculated plants and for all herbicide treatments, plants were visibly dying but structurally sound. At this point the kerosene injected plants had collapsed. By 7 weeks, all of the herbicide treated plants had died.

**Figure 1:**
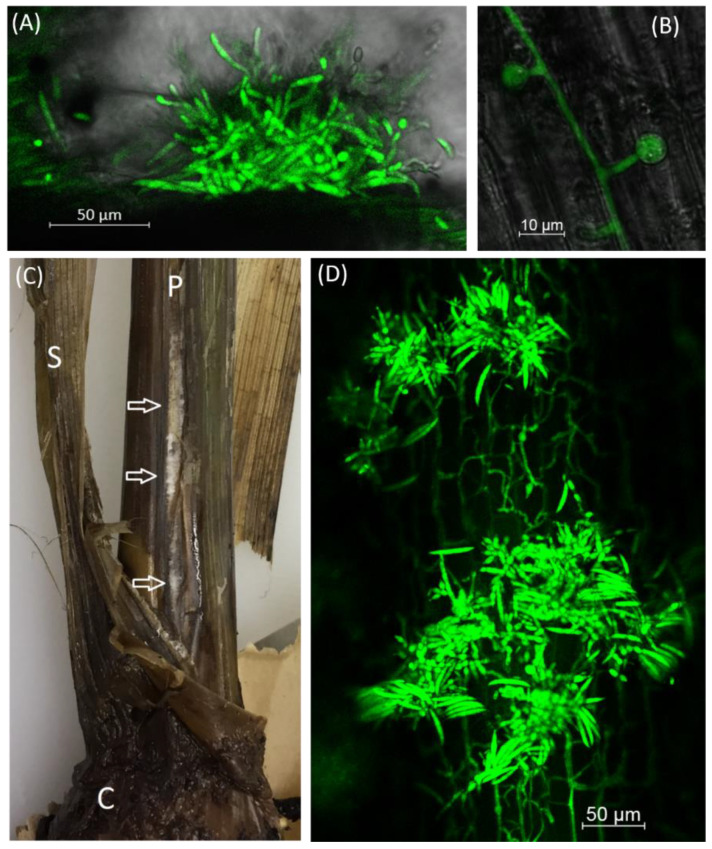
(**A**) Macroconidia arising from sporodochia on the mid-rib of the oldest erect leaf on a glyphosate injected ‘Williams’ plant 3.5 weeks after treatment with glyphosate, (**B**) Chlamydospores forming in the leaf mid-rib of an herbicide then fungicide sprayed plant (6 weeks after inoculation), (**C**) Signs of GFP *Foc* colonising the outer leaf sheath of a pseudostem of an herbicide sprayed, inoculated ‘Cavendish’ plant. White mycelia visible in the split pseudostem are highlighted with white arrows. C—corm, P—pseudostem, S—senescent leaf (16 weeks after inoculation) and (**D**) Sporodochia on the stem of a GFP *Foc* inoculated plant drenched with fungicide and then sprayed with herbicide (16 weeks after inoculation).

Sectioning and subsequent examination using scanning confocal microscopy of the corm and leaf mid-rid of the oldest erect leaf was undertaken for these plants. Within the corm tissue there was little apparent evidence of colonisation by the GFP *Foc*; non-decayed corm pieces were then taken for subsequent sectioning. However, some sections of the mid-rib of old leaves of the different herbicide treated plants showed extensive colonization by the GFP *Foc*. For the kerosene treated plant, hyphae were observed colonising the surface of the outer mid-rib while the atrazine, glyphosate and paraquat/diquat treated plants exhibited the presence of hyphae and also, microconidia and macroconidia (Figure 1A, Table 4). Although, chlamydospores were not readily found, they were seen in the corm of the kerosene treated plants and on an outer senescing leaf of the lower portion of the pseudostem of one replicate of the water injected inoculated plant.

Following plating of tissue pieces onto WA, GFP *Foc* was recovered in culture from the corm of all of the inoculated plants and from the mid-rib of the herbicide treated inoculated plants (Table 5). The GFP *Foc* isolate was not recovered from the non-inoculated controls.

### 3.2. Effect of Foliar Fungicide Application on Foc-Infected Plants Sprayed with Glyphosate

Regardless of chemical treatment regime, there was variable colonisation of tissue but generally where there was necrotic tissue, it was common to have colonisation by the GFP *Foc* (Figure 1B). The fungal structures identified are summarised in Table 6 indicating that hyphae were found in most tissue types with different treatments but that in these samples the incidence of sporulation was low and no sporodochia were observed. There was no evidence of GFP *Foc* colonisation on sections taken from non-inoculated control plants.

The GFP *Foc* isolate was consistently recovered from the corm of all inoculated plants whether they were treated with a chemical application or not (Table 7). However, GFP *Foc* isolate recovery from the mid-rib of inoculated plants was at an apparent lower incidence than in the corm and only in plants that had received some chemical treatment. Recovery from the roots was also at a relatively low level. The GFP *Foc* isolate was not recovered from any of the non-inoculated (control) plants.

### 3.3. Effect of Fungicide Drench Application on Foc-Infected Plants Sprayed with Glyphosate

Three weeks after inoculation the herbicide treated and fungicide (applied as a drench) then herbicide treated plants had started to senesce, leaves were yellowing on edges and wilting but plants had not died, younger leaf mid-ribs were still green. The plants which had not been treated with herbicide remained green. There were no distinct differences in appearance between the inoculated control (non-chemical treatment) and the drench applied fungicide only treated plants. When cut open, the lower portions of the corms from inoculated plants were brown and decayed, symptoms characteristic of *Foc* infection, regardless of whether chemicals had been applied or not. Whereas the corms of the non-inoculated controls were white with no evidence of decay.

Examination of the leaf sheaths, revealed colonisation, in the form of hyphal mats of the GFP *Foc*, visible using a hand lens, on the outer side of leaf sheaths close to the base of the pseudostem on one out of the three herbicide treated plants (Figure 1C). Likewise, one of the three drench applied fungicide then herbicide treated plants showed a similar hyphal mat on the outer leaf sheath. Sections were cut from these samples and examined using the confocal microscope. On the sections of the leaf sheath of the herbicide treated plant, microconidia were present while on the fungicide then herbicide treated plant, sporodochia were evident (Figure 1D).

In the sections of the corm pieces extensive colonisation was seen in association with decayed tissue. This extensive colonisation was particularly apparent on the herbicide treated and fungicide then herbicide treated plants in the form of hyphae and a few chlamydospores (herbicide treated sections) and micro- and macroconidia and hyphae (herbicide then fungicide treated). Whereas sparse hyphae were found in the inoculated control and fungicide treated corms.

The GFP *Foc* was recovered from at least one replicate from the roots or corm of the inoculated control, fungicide drenched, herbicide sprayed and fungicide drenched then herbicide sprayed plants (Table 8). The only treatment where the GFP *Foc* was recovered from the mid-rib was for one replicate of the fungicide drenched then herbicide sprayed treatment. The GFP *Foc* was not recovered from any of the non-inoculated plants.

## 4. Discussion

In Queensland, Australia, Fusarium wilt Tropical Race 4 has an extremely restricted distribution and is subject to tight quarantine controls [6]. To assist with the development of quarantine protocols, including the best method to dispose of infected plants, a good understanding of the epidemiology of the disease is required. We undertook glasshouse studies with the GFP- transformed isolate of *Foc* Subtropical Race 4 to examine the potential production of inoculum following treatment of infected plants with herbicide and/or fungicide.

When inoculated plants were treated with the herbicides glyphosate, atrazine or paraquat/diquat, the GFP *Foc* Subtropical Race 4 readily colonised the senescing banana plant tissue with hyphae and produced micro- and macroconidia. The inoculated control plants assessed at the same time points as the herbicide treated plants, were not extensively colonised, producing only a few chlamydospores. Warman and Aitken [8] demonstrated *Foc* readily colonises and sporulates on naturally senescent banana tissue, our observations indicate the application of herbicides hastens the process. It would be interesting to undertake time course experiments on inoculated plants to determine how much faster *Foc* can colonise herbicide treated versus those not treated with herbicide.

The fungicide propiconazole was applied alone and in combination with the herbicide glyphosate to examine the effect of the fungicide on subsequent sporulation of the GFP *Foc*. The first trial with propiconazole was as a foliar application; this reflected current practice in Australia where propiconazole is registered for use as a foliar spray to control of leaf spot diseases. This treatment had no effect on the sporulation of the Fusarium fungus; the fungus was still able to colonise the plant and produce hyphae, macro- and microconidia and chlamydospores. In the second trial a soil-drench was used with the aim that the xylem-mobile propiconazole [15] would be translocated into the corm of the plant, however this treatment was also ineffective at decreasing sporulation of the GFP *Foc*.

In these studies, propiconazole was chosen for its xylem-mobile property [15] given that initial *Foc* infections have been shown to be contained to the xylem [8]. Propiconazole had been shown previously to reduce the symptoms of Fusarium wilt when applied as a root dip prior to inoculation in glasshouse trials [12], presumably allowing movement of the chemical within the xylem prior to *Foc* colonisation. However, in this study, where propiconazole was applied after apparent colonisation, it was not effective in preventing sporulation of the pathogen. The ability of *Foc* to colonise and effectively sporulate on dead tissue as well as its xylem colonizing abilities [8] makes its control particularly problematic. To be effective, fungicide applications will need to be able to diffuse to areas of the plant where xylem transport has been compromised. Transport within the xylem can be inhibited either by the production of tyloses in a defence response by the by the infected plant and/or by the presence of the pathogen blocking the xylem as demonstrated by Warman and Aitken [8]. Once plant tissue dies, whether induced by herbicides or not, ability for fungicides to be translocated to the infected tissue is negated.

This is compounded by the ability of *F. oxysporum* in general, to persist as an effective saprophyte on killed plant tissue, thus increasing the inoculum potential. This phenomenon is apparent with other members of the *Fusarium* genus such as with the wheat crown rot fungus *F. pseudograminearum*, where inter-seasonal carryover of inoculum is enhanced when zero-till practices are deployed [16]. The subsequent reduction in the rate of plant residue degradation leads to an increase in inoculum potential. Perhaps a shift toward the use of competitive saprophytic colonisers may provide a means of reducing the proliferation of Fusarium on decayed and decaying plant tissue. This practice is used in forestry where a rapid colonising wood rot species such as *Phlebiopsis (Peniophora) gigantea* is applied to tree stumps to prevent establishment and spread of the pathogenic *Heterobasidion annosum (Fomes annosus)* forming an infection focus within a forestry plantation [17].

The ability of *Foc* to colonise uninfected banana plants or tissue at different stages of senescence should be explored. Pittaway et al. (1999) were able to recover *Foc* into culture from excised, previously uninfected banana root tips that had been placed in soil that was infested with *Foc* Race 1 and similarly in soil infested with Subtropical Race 4 [18]. This implies that, *Foc* is able to colonise excised fresh banana tissue. However, determination as to whether *Foc* is able to colonise more senescent banana plant tissue is important given current management where healthy banana plants adjacent to a TR4 incursion in a plantation are treated with herbicide. Such a practice could potentially increase *Foc* inoculum and persistence.

Whilst the isolate we worked with was Sub-tropical Race 4, there is no known reason so far to indicate that these findings would differ for Tropical Race 4.

There is still much to be done to determine how to best decrease potential inoculum when destroying infected plantations, but we have demonstrated that plants affected by Fusarium wilt treated with herbicides remain as sources of pathogen inoculum in the field.

## Figures and Tables

**Table 1:** Herbicide treatments applied by injection into stems of ‘Cavendish’ banana plants at 6 weeks post inoculation of GFP *Foc*.

GFP *Foc* Treatment	Herbicide Treatment	Rate of Herbicide Application ^b^
Inoculated	Glyphosate (360 g/L a.i.^a^)	16.67 mL/100 mL
Inoculated	Paraquat/Diquat (135 g/L/115g/L a.i.)	8.89 mL/100 mL
Inoculated	Atrazine (900 g/kg a.i.)	0.29 g/100 mL
Inoculated	Kerosene	Non-diluted
Inoculated	Water	-
Non-inoculated	-	-

^a^ a.i.—active ingredient as indicated by manufacturer. ^b^ Rate applied as based on the registered label spot spray concentrations of the various herbicides which had been scaled similarly to the glyphosate concentration. The injection rate of glyphosate for destruction of TR4 infected plants is 27.78 times the registered spot spray rate, therefore plants were injected with 27.78 times the spot spray rate for paraquat/diquat and atrazine. The volume used for 1 m tall plants is 5 mL therefore 1.6 mL was used for the 0.3–0.4 m tall glasshouse grown plants. Injection with non-diluted kerosene was used previously by banana growers to de-sucker banana plants.

**Table 2:** Herbicide and fungicide treatments applied with a hand-held atomiser to GFP *Foc* inoculated ‘Cavendish’ banana plants.

GFP *Foc* Treatment	Chemical Application Treatment ^a^
Inoculated	Fungicide (propiconazole 105 g/L a.i.) applied at 3 weeks after inoculation
Inoculated	Herbicide (glyphosate 360 g/L a.i.) applied at 4 weeks after inoculation
Inoculated	Fungicide applied at 3 weeks after inoculation followed by herbicide 4 weeks after inoculation
Inoculated	Herbicide applied at 4 weeks after inoculation followed by fungicide 5 weeks after inoculation
Inoculated	-
Non-inoculated	-

^a^ Propiconazole was applied at the label rate of 0.736 mL/L and glyphosate was applied at the spot spray label rate of 15 mL/L.

**Table 3:** Timing of fungicide drench and herbicide spray to GFP *Foc* inoculated ‘Cavendish’ banana plants.

Treatment Name	Timing of Herbicide or Fungicide Treatment
Fungicide alone	Fungicide applied as a drench 11 weeks after inoculation
Herbicide alone	Herbicide application 13 weeks after inoculation
Fungicide then herbicide	Fungicide applied as a drench 11 weeks after inoculation followed by herbicide application 2 weeks later
Inoculated control	-
Non-inoculated control	-

**Table 4:** GFP *Foc* fungal structures identified in sections of mid-rib tissue from the throat of banana plants subject to different herbicide injections. The table provides a summary of findings from three plants from each treatment (harvested 3, 3.5 and 7 weeks post treatment). At least six sections were examined from every plant. ‘+’ indicates structures were found on at least one section and indicates relative abundance across the three plants on the sections where found, ‘−’ indicates structures were not seen on any of the six sections cut for each of the three plants from each treatment.

	Fungal Structures Identified on Mid-Rib Sections				
Treatment	Hyphae	Sporodochia	Microconidia	Macroconidia	Chlamydospores
**Glyphosate**	+++	++	++	+++	−
**Paraquat** **/diquat**	+	−	+++	+++	−
**Atrazine**	+++	−	++	+	−
**Kerosene**	+	−	−	−	−
**Water injected**	−	−	−	−	−
**Non- inoculated**	−	−	−	−	−

**Table 5:** Re-isolation of the GFP *Foc* from inoculated banana plants injected with a range of herbicides. Isolations were performed on three plants per treatment with three pieces of tissue taken from throat mid-rib and four from corm per plant. The number of plants out of three from which the GFP *Foc* isolate was recovered is reported.

	Herbicide Treatment					
	Glyphosate	Paraquat /Diquat	Atrazine	Kerosene	Water Injected	Non-Inoculated
**Corm**	2/3	2/3	3/3	2/3	3/3	0/3
**Mid-rib**	1/3	2/3	1/3	1/3	0/3	0/3

**Table 6:** Fungal structures identified on sections from herbicide and fungicide sprayed GFP *Foc* inoculated plants harvested 6 weeks after inoculation. Three plants examined per treatment with six sections cut from each plant part. ‘+’ indicates structures were found on at least one section and indicates relative abundance across the three plants on the sections where found, ‘−’ indicates structures were not seen on any of the six sections cut for each of the three replicate plants.

Fungal Structures * Identified on Roots (R), Corm (C) and Mid-Rib (M) Sections												
	Hyphae			Microconidia			Macroconidia			Chlamydospores		
	R	C	M	R	C	M	R	C	M	R	C	M
Inoculated → fungicide	+	+	+	−	−	−	−	−	−	−	−	−
Inoculated → herbicide	−	+++	+	−	−	−	−	++	−	−	+	+
Inoculated → fungicide→ herbicide	−	+	++	−	+	−	−	+	−	−	++	−
Inoculated → herbicide→ fungicide	++	−	++	−	−	−	−	−	−	−	−	+
Inoculated control	+	+	+	−	−	−	−	−	−	+	+	−
Non-inoculated	−	−	−	−	−	−	−	−	−	−	−	−

* Sporodochia were not observed.

**Table 7:** Re-isolation of the GFP *Foc* from inoculated plants treated with glyphosate (herbicide) and/or propiconazole (fungicide). Isolations were performed from roots, corms and stems and the numbers in each column are the number of replicates (out of 3) from which the GFP *Foc* was recovered. For each plant, three pieces of tissue were taken each from the roots, corms and mid-rib for plating. Plants were harvested 6 weeks after inoculation.

Treatment	Roots	Corm	Mid-Rib
Inoculated, fungicide treated	0/3	3/3	1/3
Inoculated, herbicide treated	1/3	3/3	1/3
Inoculated, fungicide treated then herbicide treated	2/3	3/3	1/3
Inoculated, herbicide treated then fungicide treated	2/3	3/3	0/3
Inoculated control	1/3	2/3	0/3
Non-inoculated	0/3	0/3	0/3

**Table 8:** Re-isolation rates from fungicide drenched and herbicide sprayed GFP *Foc* inoculated plants showing the number of replicates out of three from which the GFP *Foc* was recovered. For each plant three pieces of tissue from roots and mid-ribs, and four from corms were plated. Plants were harvested 16 weeks after inoculation.

Treatment	Roots	Corm	Mid-Rib
Fungicide	3/3	1/3	0/3
Herbicide	3/3	3/3	0/3
Fungicide then herbicide	3/3	3/3	1/3
Inoculated	2/3	1/3	0/3
Non-inoculated	0/0	0/0	0/0

## Data Availability

The data presented in this study are available on request from the corresponding author. The data are not publicly available due to file size of scanning confocal microscopy images.

## References

[1] Ploetz R.C. (2015). Management of Fusarium wilt of banana: A review with special reference to tropical race 4. Crop Prot..

[2] Pegg K.G., Coates L.M., O’Neill W.T., Turner D. (2019). The epidemiology of Fusarium wilt of banana. Front. Plant Sci..

[3] Maryani N., Lombard L., Poerba Y.S., Subandiyah S., Crous P.W., Kema G.H.J. (2019). Phylogeny and genetic diversity of the banana Fusarium wilt pathogen *Fusarium oxysporum* f. sp. *cubense* in the Indonesian centre of origin. Stud. Mycol..

[4] Lombard L., Sandoval-Denis M., Lamprecht S.C., Crous P.W. (2019). Epitypification of *Fusarium oxysporum*—clearing the taxonomic chaos. Persoonia.

[5] Garcia-Bastidas F., Quintero-Vargas C., Ayala-Vasquez M., Seidl M., Schermer T., Santos-Paiva M., Noguera A.M., Aguilera-Galvez C., Wittenberg A., Hofstede R. (2019). First report of Fusarium wilt Tropical Race 4 in Cavendish bananas caused by *Fusarium odoratissimum* in Colombia. Plant Dis..

[6] (2019). Queensland Biosecurity Manual, Version 12.0.

[7] Sequeira L. (1963). Effect of urea applications on survival of *Fusarium oxysporum* f. *cubense* in soil. Phytopathology.

[8] Warman N.M., Aitken E.A.B. (2018). The Movement of *Fusarium oxysporum* f.sp. *cubense* (Sub-Tropical Race 4) in Susceptible Cultivars of Banana. Front. Plant Sci..

[9] Youssef B.A., Heitefuss R. (1982). Side-effects of herbicides on cotton wilt caused by *Fusarium oxysporum* f. sp. *vasinfectum* I. Effect of herbicides on fungal growth and wilt incidence of cotton plants. J. Plant Dis. Prot..

[10] Lèvesque C.A., Rahe J.E., Eaves D.M. (1987). Effects of glyphosate on *Fusarium* spp.: Its influence on root colonisation of weeds, propagule density in the soil and crop emergence. Can. J. Microbiol..

[11] Sanogo S., Yang X.B., Scherm H. (2000). Effects of herbicides on *Fusarium solani* f. sp. *glycines* and development of sudden death syndrome in glyphosate-tolerant soybean. Phytopathology.

[12] Nel B., Steinberg C., Labuschagne N., Viljoen A. (2007). Evaluation of fungicides and sterilants for potential application in the management of Fusarium wilt of banana. Crop Prot..

[13] Australian Pesticides and Veterinary Medicines Authority Online Services Portal. https://portal.apvma.gov.au/.

[14] Fungicide Resistance Action Committee FRAC Code List ©*2020: Fungal Control Agents Sorted by Cross Resistance Pattern and Mode of Action. https://www.frac.info/knowledge-database/knowledge-database.

[15] Ploetz R.C., Konkol J.L., Pérez-Martínez J.M., Fernandez R. (2017). Management of laurel wilt of avocado, caused by *Raffaelea lauricola*. Eur. J. Plant Pathol..

[16] Kazan K., Gardeiner D.M. (2018). Fusarium crown rot caused by *Fusarium pseudograminearum* in cereal crops: Recent progress and future prospects. Mol. Plant Pathol..

[17] Rishbeth J. (1963). Stump protection against *Fomes annosus* III: Inoculation with *Peniophora gigantea*. Ann. Appl. Biol..

[18] Pittaway P.A., Nasril N., Pegg K.G. (1999). Soil receptivity and host–pathogen dynamics in soils naturally infested with *Fusarium oxysporum* f. sp. *cubense*, the cause of Panama disease in bananas. Aust. J. Agric. Res..

